# Leisure time sports and exercise activities during the COVID-19 pandemic: a survey of working parents

**DOI:** 10.1007/s12662-021-00730-w

**Published:** 2021-06-30

**Authors:** Michael Mutz, Anne K. Reimers

**Affiliations:** 1 Institute of Sport Science, Justus-Liebig-University Gießen, Kugelberg 62, 35394 Gießen, Germany; 2 Department of Sport Science and Sport, Friedrich-Alexander-University Erlangen-Nuremberg, Gebbertstraße 123b, 91058 Erlangen, Germany

**Keywords:** Public health, Social inequality, Gender inequality, Leisure, Physical activity

## Abstract

Many working parents experienced a double burden of fulltime employment and increased childcare obligations during the COVID-19 (coronavirus disease 2019) pandemic. This paper explores how this twofold burden affected leisure time sports and exercise (LTSE). Following a gender inequality perspective, it is assumed that the level of LTSE of working mothers are more negatively affected by the pandemic than LTSE levels of working fathers. Using the nation-wide representative SPOVID survey, the paper analyses data of all respondents in fulltime employment (*N* = 631). Data collection took place in October and November 2020 in collaboration with Forsa, a leading corporation for public opinion polls in Germany. Results show that the pandemic led to a reduction of LTSE levels, but with considerable variation between working mothers and fathers. Fulltime working mothers reduced their LTSE by a substantial margin (54 min per week), but not working fathers. It is concluded that the double burden of work demands and childcare duties in the pandemic was largely shouldered by mothers, who then faced greater difficulties to remain active.

## Introduction

The COVID-19 (coronavirus disease 2019) pandemic has affected social activities and public life around the globe with Germany being no exception. In the first wave of COVID-19 infections, peaking in March and April 2020, German authorities reacted with a lockdown of public infrastructure. These containment and mitigation policies included the closing of retail shops, restaurants, museums, schools, childcare facilities as well as leisure and sports infrastructure. In this period, the German Federal States established home schooling as a substitute for in-person learning, i.e. schools provided written instructions and worksheets, but parents were supposed to supervise the daily learning tasks of their children. In December 2020, in the second wave of COVID-19 infections, many federal states again closed schools and renewed their home schooling policy.

Among Germany’s 41.5 million households (Federal Statistical Office, 2021) are 6 million where at least one child under the age of 12 is resident and in 4 million of these both parents are employed (Müller, Samtleben, Schmieder, & Wrohlich, 2021). Hence, two thirds of all families experienced a double burden in the pandemic resulting from the closing of schools and childcare facilities. These families had to manage the demands of fulltime jobs as well as additional duties relating to care, supervision of distance learning, and structuring daily routines for their children and other family members. Consequently, parents generally felt constrained by pandemic related mitigation measures and a substantial proportion of them reported more distress, negative mood, parenting-related exhaustion and a worsening of their mental health (Gassman-Pines, Oltmans Ananat, & Fitz-Henley, 2020; Marchetti et al., 2020; Patrick et al., 2020).

However, studies point to gender differences regarding this double burden: The majority of mothers, but only a minority of fathers increased the time spent with childcare and supervision of home schooling, so that care arrangements became more unequal between genders in the pandemic (Kulic, Dotti Sani, Strauss, & Bellani, 2021; Zoch, Bächmann, & Vicari, 2021). Studies also show that mothers felt more often exhausted than fathers (Ohlbrecht & Jellen, 2021) and were less satisfied with work and family life during the lockdown (Hipp & Bünning, 2021; Möhring et al., 2021).

A possible remedy for pandemic-related strains and frustrations is sport and exercise. Beyond their general benefits for various aspects of physical health (Rhodes, Janssen, Bredin, Warburton, & Bauman, 2017), sport and exercise activities are also associated with positive mood and high levels of life satisfaction (Dolan, Kavetsos, & Vlaev, 2014; Mutz, Reimers, & Demetriou, 2020). Scholars also regard a regular involvement in sports as a buffer of stress, facilitator for coping and source of resilience (Gerber & Pühse, 2009; Klaperski, 2018). Research from the COVID-19 pandemic shows that individuals who maintained or intensified physical activity, exercise or sports reported higher levels of mood and well-being (Blom et al., 2021; Brand, Timme, & Nosrat, 2020). In addition, Canadian women who reduced physical activity reported significantly lower levels of social, emotional and psychological well-being (Nienhuis & Lesser, 2020).

However, the level of sporting activity among adults has decreased during the pandemic in many countries around the globe (e.g. Brand et al., 2020; Caputo & Reichert, 2020; Constandt et al., 2020; Mutz & Gerke, 2021; Sport England, 2020). Due to the closing of sports facilities and sports infrastructure, many sports-related routines and habits were disrupted. In Germany, for instance, roughly one third of the population reduced or stopped sports activities during the lockdown, whereas only a minority of 6% intensified their activities (Mutz & Gerke, 2021). International data also show that sports and exercise activities often became shorter (regarding their duration) and lighter (regarding their intensity) compared to the prepandemic period (Brand et al., 2020). Hence, in a time of uncertainty and strain, one of the activities that provide pleasant experiences was hard to maintain. In particular, working parents are likely to having reduced or omitted sport and exercise activities because of the additional childcare obligations and supervision of home schooling and thus a scarcity of available time. In line with these considerations, a German study on physical activity during the pandemic has shown that individuals living with younger children (< 6 years) at home have a significantly smaller chance to fulfil the physical activity recommendation launched by the World Health Organization (WHO) (Maertl et al., 2021).

This paper analyses the leisure time sport and exercise (LTSE) levels of working parents and respective changes during the COVID-19 pandemic. Firstly, we investigated whether parents in fulltime employment reduced their LTSE levels during the pandemic (*Research Question #1*). Reductions among working parents are compared with other fulltime workers without childcare duties to assess if reduction are stronger among working parents. Secondly, a gender inequality perspective is applied, assuming that reductions of LTSE are stronger among working mothers than among working fathers (*Research Question #2*). By answering these two questions, this study gives first clues on how the pandemic has changed sporting behaviours of working parents and affected gender inequalities in the domain of sports.

## Methods

### Study design

The present study “Examining physical activity and sports behaviour in the face of COVID-19 pandemic” (SPOVID) is based on a large-scale, cross-sectional survey design. A sample, representing the German population (≥ 14 years, *N* = 1508), was questioned using computer-assisted web interviewing. The survey was integrated into an existing nation-wide online panel administered by FORSA, a German organization for public opinion polling and panel research. A specific feature of this panel is that all participants are recruited offline. For the recruitment, FORSA makes use of random digit dialling, i.e. randomly generated telephone numbers, including fixed line and mobile telephone numbers, which most likely leads to a random sample representing the German residential population (Häder, 2015). In contrast to many online recruited online access panels, the recruitment approach used here assured that the sample adequately represents those population segments with a greater distance to online media.

Data collection took place between October 16 and November 3, 2020. In Germany, sports infrastructure closed on November 1, 2020 due to an accelerated spread of the coronavirus. Hence, this survey took place right before the second nationwide lockdown of leisure facilities. Respondents were invited via email and were able to answer the survey on their computer, tablet or mobile phone. Overall, 1508 individuals completed the survey.

As this analysis is concerned with the interplay of fulltime work and childcare duties/supervision of home schooling during the pandemic, only respondents in fulltime employment, based on their self-report, are selected (*N* = 631). These respondents have a mean age of 43.1 years (standard deviation [SD] = 12.6). They include 221 women (35.1%) and 409 men (64.9%). Moreover, 181 of them (28.8%) indicated having at least one child (< 18 years) living in their household (among them 45 women and 136 men). A more detailed sample description is shown in Table 1.

**Table 1:** Sample characteristics

	Full sample (*N* = 1508)		Full-time workers (*N* = 631)	
	*N*	*%*	*N*	*%*
*Gender*				
Male	739	49.0	409	64.9
Female	768	51.0	221	35.1
*Child(ren) at home*				
Yes	395	26.3	181	28.8
No	1106	73.7	447	71.2
*Age*				
14–29	310	20.5	125	19.7
30–44	315	20.9	197	31.2
45–59	396	26.3	264	41.9
60+	486	32.3	46	7.2
*Educational level*				
Upper secondary	610	40.9	294	46.8
Medium secondary	477	32.0	234	37.3
Lower secondary	403	27.1	100	16.0
*Net income* (€/month)				
< 1000	187	13.8	11	2.0
1000–1999	332	24.5	119	21.0
2000–2999	387	28.6	191	33.6
3000–3999	211	15.6	109	19.2
4000–4999	113	8.3	65	11.4
> 5000	124	9.2	73	12.9
*Migration status*				
Yes	161	10.7	67	10.6
No	1346	89.3	563	89.4
*Size of residency*				
Urban area	552	36.7	265	42.0
Mid-size town	526	35.0	175	27.7
Rural area	424	28.2	191	30.3
*COVID-19 incidence*				
“At risk” region	946	64.1	413	66.6
Not “at risk”	529	35.9	207	33.4

All participants provided written consent to be contacted via email for online surveys and took part in the survey voluntarily. The study and its procedures received approval from the ethics commission of the Friedrich-Alexander-University Erlangen-Nuremberg (Reg. 387_20B).

### Measures

#### Leisure time sport and exercise

Respondents indicated their involvement in leisure time sport and exercise (LTSE) in hours per week (‘How much time did you play sport or exercise in your leisure time’) on an 8‑point rating scale with the following answer categories: 1 = ‘did not exercise or play sports’, 2 = ‘less than 1 h’, 3 = ‘about 1 h’, 4 = ‘about 2 h’, 5 = ‘about 3–4 h’, 6 = ‘about 5–6 h’, 7 = ‘about 7–14 h’ and 8 = ‘15 h or more’. We recoded the values for best capturing the weekly time of LTSE. Given that questions relating to a concrete activity with a clear anchor point have a high recall accuracy (Hipp, Bünning, Munnes, & Sauermann, 2020) the question was asked twice: first, with referral to ‘the last week’, i.e. a week during the pandemic in October 2020, and second, with referral to the time ‘before the Corona pandemic started’. These answers were then used to estimate the pandemic-related change in LTSE.

#### Sociodemographic variables

We included sociodemographic variables that relate to sports participation in Germany into the analysis (Breuer, Hallmann, & Wicker, 2011; Nobis & El-Kayed, 2019; Reimers et al., 2019): age (in years), gender, educational level (measured by school-leaving qualifications), net income (in categories from ‘no income’ up to > 5000 € per month), migration status (1st and 2nd generation immigrants) and residency in an urban or rural area. Moreover, we asked respondents whether their administrative district (county or city) had a 7-day incidence rate per 100,000 inhabitants of > 50 (so-called “at-risk region”) or not.

### Analytical approach

We documented the mean level of self-reported LTSE before the pandemic and during the pandemic (in minutes per week) as well as the change in LTSE. Moreover, we made up four distinct groups based on LTSE change patterns: “Reducers” are individuals who were involved in LTSE before the pandemic but reduced their activity level during the pandemic by more than 60 min/week. “Maintainers” are respondents who reported a similar level of LTSE (±60 min/week) before the pandemic and during the pandemic. “Intensifiers” are those individuals who increased their activity level during the pandemic by more than 60 min/week compared to the prepandemic period. A fourth group of “inactive individuals” includes all who reported no LTSE before the pandemic as well as during the pandemic. The average changes in the level of LTSE are reported for the workforce in general and in relation to gender and child(ren) at home (*Research Question 1*). Linear regression models were then calculated with the change of LTSE levels as the dependent variable. These analyses can show associations between sociodemographic variables and the change in LTSE levels. In these models, the main effects of gender and children at home were included as well as the interaction of these two variables. The interaction shows if changes differ between working mothers and working fathers (*Research Question 2*). All analyses were conducted with IBM SPSS 25.

## Results

On average the respondents in fulltime employment reported a lower level of LTSE during the pandemic (*M* = 105 min, SE = 6.5) compared to the prepandemic period (*M* = 127, SE = 6.7). Hence, the mean reduction of LTSE was 21 min (Table 2). Findings regarding individual changes further show that 19.7% of fulltime workers reduced LTSE levels compared to the prepandemic period, 44.5% maintained their prepandemic level, and 6.8% intensified LTSE. The rest (29.0%) did not engage in LTSE before or during the pandemic and can thus be considered inactive.

**Table 2:** Self-reported changes in sports and exercise activities among fulltime workers

	Sports and exercise activities (min/week)							
	*M1*	*M2*	*Diff*	*p*	Inact (%)	Red (%)	Main (%)	Int (%)
*Fulltime workers*	126.6	105.3	−21.3	< 0.01	29.0	19.7	44.5	6.8
(*N* = 631)	(6.7)	(6.5)	(5.5)					
Males	131.1	116.4	−14.7	0.03	28.7	17.4	47.4	6.5
(*N* = 409)	(8.6)	(8.9)	(7.0)					
Females	118.4	84.8	−33.6	< 0.01	29.6	23.9	39.2	7.3
(*N* = 221)	(10.5)	(8.2)	(9.0)					
Child(ren) at home: yes	131.8	112.7	−19.0	0.11	26.3	18.2	48.0	7.5
(*N* = 181)	(12.8)	(11.6)	(11.8)					
Child(ren) at home: no	124.6	102.3	−22.2	< 0.01	30.1	20.3	43.1	6.5
(*N* = 447)	(7.8)	(7.8)	(6.1)					

*M1* mean level before the pandemic, *M2* mean level during the pandemic, *Diff* mean difference, *p* significance of difference, *Inact* inactive respondents, *Red* reducers, *Main* maintainers, *Int* intensifiers

Male fulltime workers reported a reduction in LTSE levels from 131 to 116 min, i.e. by 15 min, on average (*p* = 0.03). Female fulltime workers reduced their level of LTSE by 34 min, from 118 min per week before the pandemic to 85 min per week during the pandemic (*p* < 0.01). Hence, on average, working women reduced their LTSE more than twice as much as working men. This finding is also illustrated by the higher proportion of “reducers” among women compared to men (24 vs. 17%; χ^2^ = 3.53; *p* = 0.06) and the lower proportion of “maintainers” among women compared to men (39 vs. 47%; χ^2^ = 4.11; *p* = 0.04).

The differentiation between fulltime workers with at least one child and fulltime workers living without children reveals quite similar patterns for both groups: Those with a child reduced LTSE levels from 132 to 113 min, i.e. by 19 min per week, on average (*p* = 0.11). Fulltime workers without a child reduced LTSE levels by 22 min, from 125 to 102 min (*p* < 0.01). The two groups do not differ significantly in their prepandemic LTSE level or in their level during the pandemic. Although both groups reduced LTSE by about 20 min, this difference is insignificant among respondents with a child due to the smaller group size and higher variance in this group. Proportions of reducing, maintaining, intensifying and inactive respondents did not differ significantly between fulltime workers living with and without children.

Multiple linear regression models show that the change in the level of LTSE among fulltime workers is hardly associated with sociodemographic variables (Table 3). In Model I, only residency in urban areas (*b* = 25.0; *p* = 0.05) is a significant predictor of pandemic-related changes of LTSE. Model II additionally includes the interaction effect of female gender and child(ren) at home and thus tests the hypothesis that working mothers reduced their LTSE to a greater degree than working fathers. Results indicate that the main effect of female gender on the pandemic-related change of LTSE is insignificant (*b* = −7.5; *p* = 0.55), but that female gender in combination with child(ren) in the household is associated with a significant and substantial decline of LTSE (*b* = −53.6; *p* = 0.03). The estimated change in LTSE levels for working mothers is roughly 54 min per week more negative than the estimated effect for working fathers.

**Table 3:** Linear regression model for the change in the level of sports and exercise activity

	Change of sports and exercise activities (min/week)					
	Model I			Model II		
	*b*	*SE*	*p*	*b*	*SE*	*p*
*Gender*						
Female (vs. male)	−20.2	10.8	0.06	−7.5	12.3	0.55
*Child(ren) at home*						
Yes (vs. no)	6.7	11.4	0.56	21.7	13.3	0.10
*Interaction*						
Female × child(ren)	–	–	–	**−53.6**	**25.0**	**0.03**
*Age (in years)*	−0.41	0.41	0.33	−0.44	0.41	0.29
*Educational level (vs. lower secondary)*						
Upper secondary	−9.2	15.2	0.55	−8.7	15.2	0.57
Medium secondary	−3.8	15.4	0.81	−2.5	15.4	0.87
*Personal net income*	0.07	0.18	0.69	0.09	0.18	0.60
*Migration status*	−24.1	16.7	0.15	−26.0	16.6	0.12
*Size of residency (vs. rural area)*						
Urban area	**25.0**	**12.5**	**0.05**	**24.8**	**12.5**	**0.05**
Mid-size town	16.6	13.5	0.22	16.6	13.4	0.22
*COVID-19 incidence*						
“At risk” region	−9.3	11.0	0.40	−10.2	11.0	0.35

Ordinary least squares regression

*N* = 621

R^2^ = 0.025

Values printed in *bold* are significant with *p* < 0.05

## Discussion and conclusion

Based on a representative survey collected in the period of the COVID-19 pandemic in Germany (October/November 2020), this paper documented some key insights regarding the LTSE of fulltime workers living with and without children. Firstly, the data presented here shows that the pandemic led to a reduction of LTSE levels. Considering adaptions of LTSE behaviours, we have shown that “reducers” by far outweigh “intensifiers” (19.7 vs. 6.8%), but that a substantial proportion of respondents was able to maintain prepandemic LTSE levels (44.5%). Findings thus complement previous studies on the impact of the COVID-19 pandemic on physical activity that also pointed to a decline of LTSE and analysed variations within the population (Brand et al., 2020; Caputo & Reichert, 2020; Constandt et al., 2020; Maertl et al., 2021; Schmidt et al., 2020).

However, German data from earlier stages of the COVID-19 pandemic (Mutz & Gerke, 2021) indicated that 31% reduced LTSE during the first lockdown in April 2020. This is a higher proportion compared to the period analysed here (October 2020). Similar results are shown by the weekly Sport England (2020) surveys that allow for assessing trends at the population level: Reductions in physical activity were more pronounced in the early stages of the pandemic and during lockdowns compared to later stages. Hence, after a first phase of reorientation people seem to adapt their behaviour continuously during the pandemic according to political regulations in force, current incidence dynamics and their personal risk perception.

Secondly, the analysis of working parents revealed that LTSE adaptations in this group were generally similar compared to fulltime workers without children at home. However, a closer look on working mothers and fathers revealed substantial gender differences: Whereas working fathers did not reduce their LTSE levels by larger margins, working mothers did so. This allows the conclusion that gender inequality in the domain of sport and exercise has increased during the pandemic. With an estimated difference of roughly 54 min, working mothers reduced their level of LTSE much more than their male counterparts. This large decline is certainly relevant for health as 54 min equal 36% of the minimum physical activity level (150 min/week), recommended by the World Health Organization (WHO) (Bull et al., 2020).

Our findings are in line with recent sociological accounts that consistently pointed out that the double burden of work and childcare duties is unequally distributed within families and that mothers shoulder the overwhelming share of this extra work (Hipp & Bünning, 2021; Kulic et al., 2021; Zoch et al., 2021). This has probably resulted in a shortage of time, which may be one of the reasons why more mothers reduced LTSE. In line with this interpretation, Canadian findings indicated that women with increased childcare duties during the pandemic report more barriers and difficulties to continue with physical activities (Nienhuis & Lesser, 2020). Moreover, scholars have also argued that work demands and stress reduce self-regulation capacity, which is crucial to initiate and maintain physical activity (Häusser & Mojzisch, 2017; Rouse, Ntoumanis, & Duda, 2013). The pandemic has likely changed work and family routines, demanding more self-regulation particularly from working parents. If this holds true, the reduction of LTSE levels among mothers may also be due to the exhaustion of self-regulatory capacity, which results from managing work and family at the same time.

This study has strengths and weaknesses: Very few studies elaborate on sports and exercise activities in the pandemic based on large-scale, representative samples. A major strength is thus the representative sample that allows for general conclusions on the German population. Studies on sports activities in the pandemic rarely use representative data, but often build on convenience samples (Caputo & Reichert, 2020). Data collection for this study took place in October/November 2020, i.e. in a period where incidence rates where rising, but schools and childcare facilities remained open. Hence, it can be assumed that the double burden of managing work and childcare was not at its peak during the time of data collection. Supposedly, the adaptation of LTSE reported here may even become more gender unequal in a lockdown period with closed schools and childcare facilities like in January 2021 in Germany. Given that Maertl et al. (2021) reported a negative effect on physical activity for individuals with younger children (< 6 years), it would be worthwhile to include the age of the child as another interaction into the analysis. It seems likely that younger children need more supervision than older ones and thus may differently impact on parents’ leisure pursuits. However, in the data analysed here, the exact age of the children was not collected. Likewise, there are no information regarding social support (e.g. from relatives, friends or neighbours) that families may have received to be able to handle work and childcare. Finally, findings hold true for the German context, but may differ in other countries depending on COVID-19 incidence rates, the strictness of containment policies put in force by national governments, approval or rejection of traditional gender role models and available childcare services.

It can be concluded from this study that the COVID-19 pandemic’s effect on LTSE levels is generally negative, but more complex than often assumed. Working mothers make up a group in which LTSE considerably declined, whereas other social groups were more easily able to maintain their activity levels during the pandemic. Besides greater exhaustion, nervous symptoms and reduced wellbeing (Ohlbrecht & Jellen, 2021), forced sedentariness may count as another negative health factor that working mothers are facing in the ongoing pandemic. Hence, gender inequality and the broader context of living and working conditions must be taken into account, when the impact of the COVID-19 pandemic is assessed.

## Declarations

All procedures performed in studies involving human participants were in accordance with the ethical standards of the institutional and/or national research committee and with the 1975 Helsinki declaration and its later amendments or comparable ethical standards. All participants provided written consent to be contacted via email for online surveys and took part in the survey voluntarily. The study and its procedures received approval from the ethics commission of the Friedrich-Alexander-University Erlangen-Nuremberg (Reg. 387_20B).
